# Effect of Host Change on Demographic Fitness of the Parasitoid, *Trichogramma brassicae*

**DOI:** 10.1673/031.010.7801

**Published:** 2010-06-28

**Authors:** Shahzad Iranipour, Nahid Vaez, Ghadir Nouri Ghanbalani, Rasoul Asghari Zakaria, Mohammad Mashhadi Jafarloo

**Affiliations:** ^1^ Department of Plant Protection, Faculty of Agriculture, University of Tabriz, Tabriz, Iran; ^2^ Department of Plant Protection, Faculty of Agriculture, University of Mohaghegh Ardabili, Ardabil, Iran; ^3^ Department of Plant Breeding, Faculty of Agriculture, University of Mohaghegh Ardabili, Ardabil, Iran; ^4^ Agriculture and Natural Resource Research Center of East Azerbaijan Province, Tabriz, Iran

**Keywords:** *Helicoverpa armigera*, factitious host, life table, host suitability

## Abstract

*Trichogramma brassicae* (Bezdenko) is the most important species of *Trichogramma* parasitoids in Iran. The cotton bollworm, *Helicoverpa armigera* (Hubner) is a polyphagous insect pest that attacks many crops including cotton, maize, soybean, tomato, etc. The bollworm egg is a suitable target for many *Trichogramma* species. Factitious hosts such as eggs of the flour moth, *Anagasta kuehniella* (Zeller) and cereal moth, *Sitotroga cerealella* (Hubner) are used for mass rearing purposes. But a problem that arises sometimes in laboratory cultures is the development of a tendency toward laboratory hosts following a few generations rearing with them. This may tend to a low efficiency on target pest in field conditions. In this study the possibility of declining efficiency of the parasitoid on target pest by developing such a preference to alternative hosts in previous generations were investigated when the flour moth or cereal moth uses as laboratory host. Two generations of *T*. *brassicae* were reared on each of the mentioned hosts and then transferred to *H*. *armigera* eggs for two further generations. The intrinsic rate of natural increase as well as other life table parameters were used for monitoring fitness of the parasitoid at successive generations. Even generations were included to determine if previously rearing host affected parasitoid performance. Results revealed that host shift from cereal moth to bollworm caused a sudden fall in population growth parameters (both intrinsic rate of natural increase and net replacement rate). Further rearing on bollworm eggs led to a relapse in both parameters. No similar effect was observed in cultures initiated with the flour moth. As a whole, cereal moth was a more suitable host than flour moth.

## Introduction

*Trichogramma* species are the most studied groups of egg parasitoids. Their worldwide use in biological control programs is due to their efficiency and to be easy for rearing under laboratory conditions (Parra and Zucchi 1997). *Trichogramma* species are used successfully against several insect pests on different crop-pest systems in more than 30 countries (Li 1994). They can be found in the majority of agroecosystems where their hosts are under control by different species. Insectpests of major crops such as corn, cotton, sugar-cane, tomato, apple, rice and stored products have been targeted (Hassan et al. 1988; Ciochia 1991; Nikonov et al. 1991; Romeis and Shanower 1996; Ebrahimi 2004). The main advantages of trichogrammatids over other natural enemies are their short generation time and an economic method of mass production (Li 1994). *Trichogramma brassicae* Bezdenko is the most important species in Iran (Ebrahimi et al. 1998). According to Shojai et al. (1998), this species is a promising control agent in integrated pest management of cotton bollworm, *Helicoverpa armigera* (Hubner) in cotton fields.

Cotton bollworm is a polyphagous species and one of the most important insect pests in several major crops such as cotton, tomato, maize, pea, soybean, alfalfa etc. (Behdad 2002; Khanjani 2005). Because of a direct damage to reproductive organs of the host plants, and high reproduction rate of the pest, injury level is high. So, its control in egg stage using the egg parasitoids will be promising.

Difficulty in providing suitable condition in laboratory and storing for subsequent use are the most important inhibitor factors in mass rearing of *Trichogramma.* Since, quality of produced wasps is affected by physical and nutritional conditions (especially laboratory hosts), quality control is required to insure their field efficiency (Bigler 1994; Van Driesche and Bellows 1996). There are many studies subject to assess the quality of *Trichogramma* species (e.g. Losey and Calvin 1995; Shirazi et al. 2006).

Alternative hosts particularly *Anagasta kuehniella* (Zeller) and *Sitotroga cerealella* (Olivier) (Flanders 1927; Stinner 1977; El-Wakeil 2007; Farazmand et al. 2007) as well as artificial diets (Grenier 1994; Heslin et al. 2005) are often used for mass-rearing of *Trichogramma* spp. A reliable rearing method is necessary in every biological control program.

An enhancement has been reported in oviposition preference of parasitoids to a special host, while they are developed in alternative hosts (van Driesche and Bellows 1996). Van Bergeijk et al. (1989) reported that host acceptance of *Trichogramma maidis* (Pintureau et Voegelé) to its natural host, European corn borer, *Ostrinia nubilalis* (Hubner) reduced following three generations reared on *A. kuehniella* eggs.

Since parasitoids are able to learn, augmentation on the same host that will encounter in nature will increase probability of successful parasitism in field. In contrast, rearing them on factitious hosts may lead to a lower performance on target host (Hoffmann et al. 2001). Contact of parasitoids with target pest before release, can improve their efficiency in field (Noldus et al. 1990). It seems that, rearing an additional generation on target pest may strengthen searching ability and enhance host acceptance.

In a preliminary evaluation of natural enemies, determining their intrinsic rate of increase (r_m_) is important because not only it does directly represents their potential as biological control agents but also determines releasing method (i.e. inoculative, seasonal inoculative or inundative) (van Lenteren 1986). The same parameter was used to compare hosts by Iranipour et al. (2009). Somewhat different method and criteria were used by El-Wakeil (2007) in a similar study for evaluating efficiency of *Trichogramma evanescens* (Westwood) on *H. armigera* reared on different factitious hosts. Parasitism rate, emergence rate, longevity, developmental time, total fecundity and sex ratio were the parameters used for similar purposes by El-Wakeil (2007), Farazmand et al. (2007), and Vaez et al. (2009). In this study it has been tried to explore necessity of habituating *T. brassicae* to its target pest before releasing on *H*. *armigera.*

## Materials and Methods

### Source of hosts

*H*. *armigera* larvae were obtained from a stock that has been rearing at Department of Plant Protection, University of Tabriz. A stock culture was established using an artificial diet according to procedures elaborated by Twine (1971) and illustrated by Singh (1982). Ten pairs of newly emerged adults were confined in a plastic cylinder (18 cm in diameter and 20 cm in height), inside which had covered with a thin layer of toilet paper. Moths were fed by 20% solution of sucrose. Eggs laid on internal paper sheet were collected daily by a soft brush.

A stock culture of *A. kuehniella* was established using cultures obtained from Department of Plant Protection, University of Tabriz. The larvae were reared on wheat flour (cultivar Omid) in 20×14×6 cm rectangular containers. Adults emerged were transferred to another cylindrical containers 18 cm in diameter and 30 cm in height. Then the containers were covered by a 50 mesh cloth net and reversed on a paper sheet. Deposited eggs were attached to a thick paper using sucrose solution and then used in experiments.

A stock culture of *S. cerealella* was brought from Agriculture and Natural Resources Research Institute of East Azerbaijan Province, Tabriz, and reared on barley (cultivar Makooyi) as Jimenez and Murgueitio (1991).

### Source of parasitoid

*T*. *brassicae* was obtained as parasitized eggs of cereal moth from Agriculture and Natural Resources Research Institute of East Azerbaijan Province, Tabriz and was maintained in glass tubes 1.5×10 cm in an incubator. They were reared separately on *A. kuehniella* and *S. cerealella* eggs for four generations before using in experiments.

### General conditions of experiments

All host cultures were held at 25 ± 2 °C, 65 ± 10% RH, and 16L: 8D photoperiod in a section of a greenhouse in Plant Protection Department of University of Tabriz. All of experiments were carried out at 25 ± 1 °C, 65 ± 5 % RH, and 16L: 8D photoperiod in an incubator (model GJ01 made by Jihad center of University of Tehran).

### Life table studies and statistical analyses

Each cohort was provided using 25 mated females of *T*. *brassicae* at the same age. For this purpose, ≤ 24 h old fertile females were kept individually in glass vials (1.5×10 cm). The females were fed with a mixture of honey (20%), and distilled water (80%). Fifty to 70 fresh eggs of *S. cerealella, A. kuehniella* and *H*. *armigera were* stuck on a paper sheet (1×5 cm), and each one was offered daily to each female until the all females were died. Those females that died due to submerging in honey droplets or those ones which injured during daily handling were excluded from the analysis. Immature data involving developmental time and survival was obtained in a separate experiment by a random selection of 60 parasitized eggs of each host. Paper sheets were removed daily and incubated under laboratory conditions, until emergence.

Life table parameters including intrinsic rate of natural increase (r_m_), finite rate of increase (λ), gross reproductive rate (GRR), net reproductive rate (R_0_), mean generation time (T) and doubling time (DT) were calculated as Carey (1993); Ebert (1999); Burden and Faires (2005). For calculating intrinsic rate of natural increase, the Euler-Lotka equation was solved with an initial amount of r_c_. Means and standard errors of population parameters were estimated using the Jackknife method (Sokal and Rohlf 1981; Meyer et al. 1986) by defining functions in excel spreadsheet. Entropy amounts (Demitrius 1978; Carey 1993) were used as a scale for determining type of survivorship curves. An entropy < 0.5 may show a convex curve, > 0.5 a concave one and just 0.5 shows a linear decrease.

### Experimental design and treatments

There were eight series of treatments in two sets of experiments. In each experiment, two initial generations devoted to alternative hosts (either *A. kuehniella* or *S. cerealella*) followed by two subsequent generations on target-pest *H*. *armigera.* Life table parameters of the parasitoid on former generations then were compared to next ones. Even generations on each host was as a control for determining role of habituation in increasing fitness of the parasitoid. A comparison also was done among hosts. Intrinsic rate of natural increase (r_m_) as well as other life table parameters considered as criteria of fitness, because they can present demographic and physiological potential of a cohort (Andrewartha and Birch 1954; Carey 1993).

The experiment was carried out as a split plot in time at the base of a randomized complete block design (RCBD). The main factor was generation (four levels) and the sub-treatment was host (two levels). As mentioned earlier two hosts were *A. kuehniella* and *S. cerealella* at commence (represents by A and S respectively). After two generations, they were shifted both to *H*. *armigera* (represents by AH and SH for those treatments which their previous host was *A. kuehniella* and *S. cerealella* respectively). In order to compare bollworm to alternative hosts, analysis was repeated once again in another respect, as hosts were considered as four levels of the first factor while two generations of each one were considered as two levels of second factor. Data analyses and comparisons of means by Duncan's multiple range test both at 95 and 99% confidence level were carried out using the SAS procedures (SAS Institute Inc.).

## Results

Significant differences were observed in major of life table parameters whether among hosts or generations (except for T among hosts). Interactions between two factors also were significant in all parameters except T. Results have been summarized in Table 1.

In both experiments shift in host was tended to a fall in GRR, R_0_, r_m_, and λ and a simultaneous rise in T and DT. The intensity and direction of differences however was unequal. Differences were highly significant (P < 0.01) in all cases except for T in *S. cerealella* whereas in *A. kuehniella* only R_0_ differed significantly (based on average of the two generations, see Table 1). The results are summarized as follows:

### Intrinsic rate of natural increase

The most crucial statistic that represents fitness of a population is r_m_. Value of this parameter ranged from 0.3340 ± 0.0120 to 0.3908 ± 0.0106 in different treatments (table 1); such a small difference was significant (df = 3, 66, F = 17.78, P < 0.01). Intrinsic rate of natural increase was significantly higher on *S. cerealella* (0.3895 ± 0.0065 as average of the two generations) than the other hosts (Table 1). Value of r_m_ was similar on *H*. *armigera* whether parasitoid has already reared on *A. kuehniella* or *S. cerealella* (0.342 and 0.348 on AH and SH respectively). On the other hand, it was more significantly (P < 0.01) in the 2^nd^ generation than the subsequent generations. Population growth rate was higher in *S. cerealella* than *A. kuehniella,* but host change caused a more considerable decline in former at 3^rd^ generation. However, the growth rate of SH exceeded AH at the 4^th^ generation again.

**Table 1.**
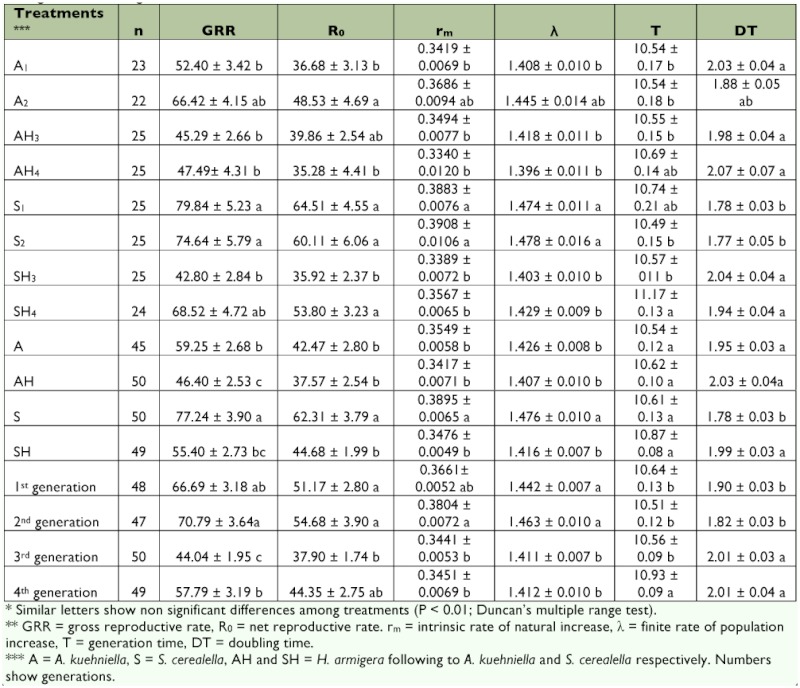
Mean ± standard error* of different demographic parameters ** of *T. brassicae* on three species of hosts in four sets of arrangements in four generations.

### Net replacement rate

R_0_ parameter shows number of daughters replaced in place of their mothers. Net replacement rate also was more significantly (P < 0.01) on *S*. *cerealella* (62.31 ± 3.79 at average) than other hosts. It ranged from 35.28 ± 4.41 to 64.51 ± 4.55 in different treatments. Difference among generations was significant (P < 0.01) too, as it was significantly less in 3^rd^ generation than both 1^st^ and 2^nd^ ones. Similar trends were observed in r_m_, R_0_, λ and GRR. Gross reproductive rate ranged from 42 to 80 females/ female/ generation in different treatments (table1).

### Mean generation time

No significant difference was observed in T among hosts. Nevertheless it was significantly longer (P < 0.01) in 4^th^ generation than in the previous generations. Value of T in all treatments ranged from 10.49 to 10.74 except for 4^th^ generation of SH (Table 1). In later treatment however, it was 11.17 ± 0.13 days at average. Regarding to above results, any change in r_m_ has been occurred due to difference in fecundity rather than generation time. Population doubled every two days (ranged from 1.77 to 2.07 in different treatments).

### Mortality distribution and survivorship curves

Survivorship curves (not shown in the article) were type I of Slobodkin (1962) in all treatments. Entropy was used as a criterion for judgment about type of curves. In all cases it was < 0.5 i.e. ranged from 0.19 to 0.31. It means that majority of mortality has been occurred at senescent. Overall immature mortality was not more than 10 % in any case (6–9 % in different treatments). The highest expectation of life was 17.98 days in SH_2_ at commence of the experiment. The lowest life expectancy at commence was 14.37 days in A_1_.

## Discussion

Intrinsic rates of population increase in all treatments of this study (0.334 to 0.391 females/ female/ day) are well in the range of some *Trichogramma* species studied by other scientists (Lu 1992; Haile et al. 2002; Pratissoli et al. 2004; Kriesemer 2004; Shirazi et al. 2008). r_m_ values were lower in Moezzipour et al. (2006, 2008) studies on *T*. *brassicae* populations of Saveh and Yazd pomegranate orchards. Relative humidity also had a considerable effect on r_m_ in their work, as it was 0.096, 0.33 and 0.23 in 45, 65 and 85 ± 5 % RH in a constant temperature i.e. 30 °C. Value of r_m_ in 65 ± 5 % RH resembles our results. Iranipour et al. (2009) also measured lower r_m_ values on *A. kuehniella* and *Plodia interpunctella* (Hubner) than the present study (0.287–0.295 and 0.209–0.218, respectively). Present differences even though small in some cases, may be owing to different sources of variations such as experimental conditions, differences in parasitoid and/or host species or populations, and analytical approaches. Such a statement may be true in the case of the other life table parameters (GRR, R_0_, λ, etc.) too.

Net reproductive rate was in the range of 35–65 females/ female/ generation in different treatments in our study. It was 62.3, 44.7, 42.5, and 37.6 in S, SH, A, and AH respectively. Obviously, higher value of this parameter in *S. cerealella* may show its suitability in comparison to other hosts. Our results are similar with Pratissoli et al. (2004) who estimated R_0_ to be 31.53 and 54.97 in *T. pretiosum* and 20.64 and 20.42 in *T. acacioi* on flour moth eggs at 25 and 30 °C respectively. Value of R_0_ was 15.85 for *T*. *chilonis* (Zhang et al. 2001), 35.16 for *Ttichogramma* sp. nr. *mwanzai* at 26 °C (Haile et al. 2002), 35.9 and 31.9 for the same parasitoid at 25 and 30 °C respectively (Lu 1992), 55.95 for *T*. *chilonis* on *C. cephalonica* at 27 °C (Kriesemer 2004) and 40.07–50.76 on *A. kuehniella* and 17.05–22.28 on *P. interpunctella* for *T*. *brassicae* at 24 °C (Iranipour et al. 2009). Excluding *T. chilonis* in Zhang et al. (2001), and *T*. *brassicae* on *P. interpunctella* in Iranipour et al. (2009) studies, other results are in high agreement to our findings. On the other hand R_0_ values were considerably lower in Moezzipour et al. (2006, 2008) studies especially in extreme temperatures and humidities (25.47 to 30.95 in median treatments and 2.43 to 13.23 at extreme treatments except for 20 °C in Saveh population that was 29.25). Differences may be due to the reasons mentioned earlier. Similar differences were observed in GRR and λ too.

Generation time is very less varying statistic than other life table parameters; however it was very sensible to small deviations, thus a small difference as low as 0.5 days (less than 5% of mean) was tend to significance. This confirms by Iranipour et al. (2009). Long generation time in SH_4_ also can be due to biases arising by rare events. T in other studies excluding Moezzipour et al. (2006, 2008) was near to the present study. It ranged from 10.8 to 11.9 days in different species under different conditions mentioned earlier as recorded by Zhang et al. (2001); Haile et al. (2002); Pratissoli et al. (2004); Kriesemer (2004). Based on our data, population was doubled every 1.77– 2.07 days in different experiments. These are shorter than values reported by other researchers (Zhang et al. 2001; Haile et al. 2002; Kriesemer 2004; Moezzipour et al. 2006; Iranipour et al. 2009). They estimated DT's from 2.18 to 4.42 days in different experiments with the species and the conditions mentioned above. Like other parameters, host and/or parasitoid species, analytical approaches, and experimental conditions may be responsible for observed differences both in DT and T.

One of the most important aspects in the present study is a sudden fall in R_0_ and r_m_ in 3^rd^ generation when the parasitoid shifted from cereal moth to bollworm. This may show an inverse effect of host change on parasitoid performance. This is true in the case of other biological parameters such as longevity, rate of emergence and total fecundity (Vaez et al. 2009). However it was observed no similar effects when alternative host was flour moth.

Further rearing on bollworm caused a relapse in both statistics at 4^th^ generation. Taking into account that *T*. *brassicae* parasitizes more eggs and grows more accelerated on cereal moth than flour moth, mass rearing on former species can be recommendable. Nevertheless, if mass rearing be relying on cereal moth, the result of inundative release in a *H*. *armigera* — *T*. *brassicae* system may be weaker than when it is base on flour moth, unless the final generation of the parasitoid be reared on bollworm rather than cereal moth. This is in contrast to El-Wakeil (2007). He stated that the wasps originated from *Sitotroga* eggs are equal in fitness to those reared from *Helicoverpa.* This is due to experimental approach and fitness criteria used which were different in his study. The order of host suitability was as *H*. *armigera* and *S. cereallela* > *A. kuehniella* > *Galleria mellonella* (L.) (El-Wakeil 2007). This may be compared to the status of the hosts in this study. Farazmand et al. (2007) also showed that *A. kuehniella* is a better host than *P. interpunctella.*

As a conclusion, it may be stated that, lack of a fortune to contact with target kairomones by egg parasitoids when they are rearing on alternative hosts (Noldus, 1989), may lead to a loss in performance when they encounter target pest for the first time in field. For example van Bergeijk et al. (1989) noticed that *T*. *brassicae* had stronger impact on *Ostrinia nubilalis* when it was reared on the same host than *A. kuehniella.* This is like *H*. *armigera* — *T. brassicae* system in this study, but does not agree to El-Wakeil (2007) results on *T. evanescens.* On the other hand Shojai et al. (1998) claimed that no similar measurement is needed when *T*. *brassicae* reared on *A. kuehniella* before releasing on *Cydia pomonella* (L.) in apple orchards. Based on the present study, this may be true in the case of similar system when bollworm be targeted rather than *C*. *pomonella.* Some strains of *Trichogramma* such as *Trichogramma dendrolimi* (Matsumura) did not lose their preference to their native host despite the rearing was carried out on a factitious host for several years as mentioned by Pavlik (1993); Lui et al. (1998); Takada et al. (2001). Hassan (1989) also reported that a few strains of *T. dendrolimi* showed a nearly equal preference between the target pests *Cydia pomonella, Adoxophyes orana* (Fischer von Röslerstamm) and the factitious host *Sitotroga cerealella* after rearing for at least two generations. The easiest way to overcome the problem is rearing final generation of parasitoid on its target pest before release. Such an action may strengthen host finding ability as well as host detection and acceptance by parasitoid (Noldus et al. 1990; van Driesche and Bellows 1996). This may be recommendable at least in the case of *T*. *brassicae* cultures on *S. cerealella.*
